# Dermatological Manifestations in Pediatric Inflammatory Bowel Disease

**DOI:** 10.3390/medicina56090425

**Published:** 2020-08-23

**Authors:** Smaranda Diaconescu, Silvia Strat, Gheorghe G. Balan, Carmen Anton, Gabriela Stefanescu, Ileana Ioniuc, Ana Maria Alexandra Stanescu

**Affiliations:** 1 Department of Pediatrics, “Grigore T. Popa” University of Medicine and Pharmacy, 700115 Iasi, Romania; turti23@yahoo.com (S.D.); ileanaioniuc@gmail.com (I.I.); 2 Clinical Department of Pediatric Gastroenterology, “St. Mary” Emergency Children’s Hospital, 700309 Iasi, Romania; 3 Gastroenterology and Hepatology Clinic, “St. Spiridon” Emergency Hospital, 700111 Iasi, Romania; balan.gheo@me.com (G.G.B.); carmen.anton@umfiasi.ro (C.A.); gabriela.stefanescu@gmail.com (G.S.); 4 Department of Gastroenterology and Hepatology, “Grigore T. Popa” University of Medicine and Pharmacy, 700115 Iasi, Romania; 5 Department of Pediatrics, “St. Mary” Emergency Children’s Hospital, 700309 Iasi, Romania; 6 Department of Dermatology, “Carol Davila” University of Medicine and Pharmacy, 050474 Bucharest, Romania; alexandrazotta@yahoo.com

**Keywords:** dermatological, pediatric, ulcerative colitis, Crohn’s disease, treatment

## Abstract

*Background and Objectives*: Over the last years, inflammatory bowel disease (IBD) has been reported on a high incidence in pediatric populations and has been associated with numerous extraintestinal manifestations, making its management a real challenge for the pediatric gastroenterologist. Dermatological manifestations in IBD are either specific, related to the disease activity or treatment-associated, or non-specific. This literature review aims to identify and report the dermatological manifestations of IBD in children, the correlation between their appearance and the demographical characteristics, the relationship between these lesions and disease activity, and to highlight the impact of dermatological manifestations on an IBD treatment regime. *Materials and Methods*: A systemic literature review was performed, investigating articles and case reports on dermatological manifestations in children with IBD starting from 2005. A total of 159 potentially suitable articles were identified and after the exclusion process, 75 articles were selected. *Results*: The most common dermatological manifestations reported in pediatric IBD are erythema nodosum and pyoderma gangrenosum. More rare cases of metastatic Crohn’s disease, epidermolysis bullosa acquisita, small-vessel vasculitis, necrotizing vasculitis, leukocytoclastic vasculitis, cutaneous polyarteritis nodosa, and Sweet’s syndrome have been reported. Oral manifestations of IBD are divided into specific (tag-like lesions, mucogingivitis, lip swelling with vertical fissures, aphthous stomatitis, and pyostomatitis vegetans) and non-specific. IBD treatment may present with side effects involving the skin and mucosa. Anti-tumor necrosis factor agents have been linked to opportunistic skin infections, psoriasiform lesions, and a potentially increased risk for skin cancer. Cutaneous manifestations such as acrodermatitis enteropathica, purpuric lesions, and angular cheilitis may appear secondary to malnutrition and/or malabsorption. *Conclusions*: The correct diagnosis of dermatological manifestations in pediatric IBD is of paramount importance because of their impact on disease activity, treatment options, and a patient’s psychological status.

## 1. Introduction

Pediatric inflammatory bowel disease (IBD) includes a series of subtypes: Crohn’s disease (CD), ulcerative colitis (UC), and IBD-unclassified (a form of colonic IBD with non-specific features, making it impossible to differentiate into colitis of CD or UC) [1].

IBD should be regarded as a disease with systemic impact, and not limited to the digestive tract. It is estimated that 6–23% of children with IBD develop extraintestinal manifestations (with a higher frequency in those older than six), either related to the disease itself or related to the medication [2,3].

In general, extraintestinal manifestations of IBD can be divided into two groups, depending on the pathophysiology. On one hand, there are IBD activity-related lesions, which have a similar immune mechanism (affecting the joints—peripheral and axial arthropathies; involving the skin—erythema nodosum, pyoderma gangrenosum, Sweet’s syndrome, aphthous stomatitis; the eye- episcleritis or uveitis) [4,5].

On the other hand, there are independent autoimmune diseases that are highly associated with IBD, such as primary sclerosing cholangitis (which has an important impact on the disease course, medication, and cancer surveillance strategies).

Cutaneous manifestations in patients with IBD are usually either specific lesions, reactivation lesions, manifestations related to nutritional malabsorption, or therapeutic regime, or they can be miscellaneous lesions [4,5]. Their diagnosis is based on the clinical characteristic features and on the exclusion of other dermatological disorders.

In the present study, we conducted a systematic review of the dermatological manifestations in pediatric IBD.

## 2. Material and Methods

**Figure 1:**
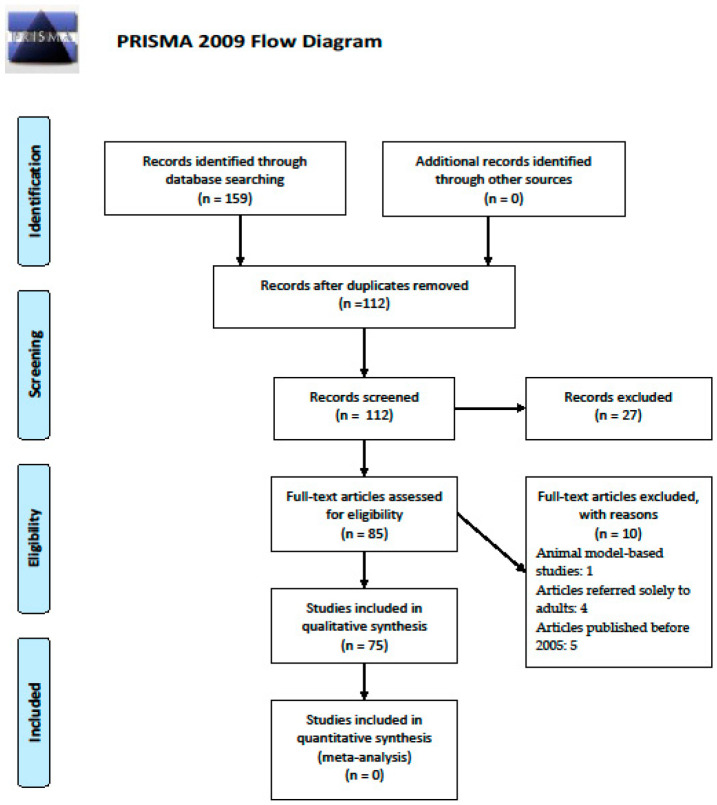
The PRISMA flow diagram, adapted from [6].

We conducted a systematic literature review starting in 2005 by consulting the PubMed, Semantic Scholar, Mendeley, and Web of Science databases for all relevant articles and case reports on extraintestinal and more specifically on the dermatological manifestations of pediatric IBD, in accordance with the PRISMA (Preferred Reporting Items for Systematic Reviews and Meta-Analyses) guidelines (Figure 1).

During this process, we used combinations of the following search terms: “dermatological manifestations in pediatric IBD”, “cutaneous manifestations in pediatric IBD”, and “skin manifestations in pediatric IBD”. 

## 3. Results

We identified a total of 159 potentially suitable articles. The exclusion criteria were: articles selected twice, animal model-based studies, articles referred solely to adults, and articles published before 2005. 

After this process, 75 articles were selected. We analyzed the reports on the cutaneous manifestations in pediatric IBD trying to point out their incidence, age of appearance, particularities, and the relation between disease activity and skin lesions.

Authors report that up to 80% of children with CD and 50% of those with UC experienced at least one extraintestinal manifestation [7].

Dermatological manifestations are seen in children with both CD and UC, their incidence varying from 10–15% [4]. They are either specific or reactivation lesions or may be related to malabsorption or drugs used in the treatment of IBD; a small group of lesions have been described in the literature concomitant to IBD but are considered miscellaneous lesions (Table 1) [3]. 

In one Tunisian study, 6 out of 14 children with IBD presented with dermatologic manifestations as follows: three children presented aphthous stomatitis, one child presented erythema nodosum, and there was one case of ulcerative skin eruption and one case of vitiligo [8]. The dermatological manifestations of pediatric IBD are reported in the literature either through small series or case reports (Table 2). 

### 3.1. Specific Lesions

Dermatological manifestations specific for IBD include mucosal and skin lesions such as fissures, fistulas, aphthous stomatitis, mucogingivitis, lip swelling, pyostomatitis vegetans, metastatic Crohn’s disease, and epidermolysis bullosa acquisita.

Based on the presence of granulomas, oral manifestations of IBD are divided into specific and non-specific. Specific oral lesions are not associated with disease activity and include indurated tag-like lesions, mucogingivitis, lip swelling with vertical fissures, aphthous stomatitis, and pyostomatitis vegetans (which has been linked to IBD activity) [9].

Studies report that oral involvement in pediatric UC patients may appear in up to one-third of the cases and usually consists of non-specific lesions. Lesions of the oral cavity may be painful and may lead to impaired oral function or to psychological misbalances due to esthetic involvement [9]. Lesions of the oral mucosa may seem more severe during a period of disease exacerbation but 30% of patients, especially of pediatric age, present active oral manifestations during IBD remission [10]. 

In a group of 1649 pediatric IBD patients, 97 children presented with extraintestinal manifestations, 21% of which were aphthous stomatitis [3]. In another retrospective study, 24% of children with IBD presented dermatological manifestations, 18% of which were aphthous stomatitis [10]. In a study from Iran, oral aphthous lesions were identified in 13% of patients with CD vs 6% of UC patients. Aphthous stomatitis has been associated with erythema nodosum [12]. 

Aphthous stomatitis is also associated with celiac sprue, HIV infection, oral herpes simplex infection, Coxsackievirus infection, Reiter’s syndrome, and Behçet’s disease [13].

Pyostomatitis vegetans is very rare in children with IBD, being reported only in a few cases. It appears as white or yellow pustules on a thickened erythematous mucosa. The pustules undergo a process of degeneration, ulceration, and suppuration, leading to a “snail track” aspect. It is usually located on the buccal mucosa, tonsillar regions, and soft and hard palate; the floor of the mouth and tongue are usually spared. In children, pyostomatitis vegetans has been associated mostly with UC, but there have been case reports where it was linked to CD [14,15]. 

Pyodermatitis vegetans, a rare dermatological manifestation of IBD, is often regarded as one of the clinical forms of pyoderma gangrenosum. It occurs mainly in skin folds on the axillary or inguinal area but can also appear on the trunk or extremities [16].

Metastatic Crohn’s Disease (MCD) is a granulomatous dermatitis characterized by the presence of non-caseation granulomatous inflammation occurring at a distance from the gastrointestinal tract. It manifests as plaques, abscesses, swelling, or fissures mainly on the arms, legs, and face [17].

MCD is rarely reported, with less than 100 cases in the literature. The age in children with MCD varies from 5 to 17 years of age and the sex incidence seems to be equal [18]. MCD has been associated with CD activity; gastrointestinal flares were identified in children with MCD within a wide variation of time after the MCD presentation (9 months to 14 years). However, literature reports show that in almost 50% of children MCD appears concomitantly with the first gastrointestinal symptoms. In asymptomatic children, a CD diagnosis is expected within months or years [18]. 

Bender-Heine et al. reported that the features of dermatological findings in MCD correlated with age—lymphedema is most commonly found in children or young adults [18]. Blasco Alonso et al. reported a group of four pediatric patients diagnosed with MCD, three of whom were diagnosed prior to the IBD diagnosis. In this lot, the lesions were distributed in the genital area in three patients and in the bilateral pretibial region in one patient [17].

Children with MCD seem to be more prone to genital lesions. Up to 85% of cases showing some degree of genital swelling with or without induration. There are pediatric cases of penis and scrotal swelling, erythematous, or ulcerative lesions in male patients and vulvar swelling or pre-clitoral masses in females [17].

Korean authors report the case of a 10-year-old girl who presented with vulvitis as the initial symptom; skin biopsy set the diagnosis of MCD [19]. Anal skin tags have been described in both adult and pediatric CD as flesh-colored, waxy, painless, raised, firm, narrow or broad anal malformations that usually appear in group and may sometimes present granulomatous tissue [20].

Keljo et al. assessed the disease course and treatment of pediatric CD patients with perianal disease. Out of 276 patients, 41 presented perianal lesions within a 30 day-interval after diagnosis (13-skin tags and fissures; 28-fistulas/abscesses). Although most of the fistulas and abscesses resolved within one year (20), eight patients presented chronic recurrent perianal disease and were more predisposed to lower body mass indices and surgical interventions. In this study, patients without fistulas presented the tendency to receive earlier prednisolone treatment; patients presenting with fistulas were treated with Infliximab with a favorable outcome after three months [21].

Epidermolysis bullosa acquisita (EBA) has been associated with IBD, especially CD, but there have been cases of UC association. Controversy exists as to whether EBA appears parallel to the disease activity and to whether it is a specific lesion at all or is found incidentally. It is estimated that 30% of patients with EBA have been later diagnosed with CD as well. Specific autoantibodies against type VII collagen (expressed in the skin, esophagus, oral, anal, and colonic mucosa) have been associated with both CD and EBA, and, with a lower frequency, with UC [29,62]. 

Authors reported that the onset of digestive symptoms usually precedes the skin-blistering, but there are cases when milder gastrointestinal symptoms were overlooked or misdiagnosed as irritable bowel syndrome [28].

### 3.2. Reactivation Lesions

Erythema nodosum (EN) is the most cited reactivation dermatological manifestation in IBD, occurring in 10–15% of people with IBD, CD in particular. In adults, EN seems to appear mostly in females (male-to-female ratio of 1:6), but the sex ratio in children seems to be 1:1 [27]. Dotson et al. reported that 2.8% of children with IBD (from a 1009 children lot) developed EN during the follow-up period of time (2.6% had CD and 0.2% had UC) [26].

EN usually evolves parallel to the IBD activity, but there have been cases where it preceded the diagnosis of IBD by up to five years. There was one reported case where a child (11-year-old girl) presenting only EN and no gastrointestinal symptomatology was later on diagnosed with active CD [25].

In most cases, treating the IBD also controls the evolution of EN; however, there are cases where systemic corticotherapy or immunosuppressive medication is indicated [25]. 

Pyoderma gangrenosum (PG) is a rare recurrent painful skin disease from the spectrum of neutrophilic dermatosis, which is a non-infectious inflammatory cutaneous disease. The typical appearance of PG consists of pustules with quick evolution into painful ulcers, with undermined violaceous borders surrounded by erythema. In 20% of patients, ulcerations may worsen on traumatized areas (because of biopsy, surgery, or cannulation) [23,24].

PG is associated with several systemic diseases, such as IBD, leukemia, and immunodeficiency (IgA deficiency and HIV positive status) [63].

The distribution of PG on the body is different in children. Usually, it is located on the extensor surfaces of the extremities; in the pediatric population, PG has been reported more often on the legs, followed by the head and the gluteal region. It has been reported in 0.3–5% of patients with IBD [4]. The authors reported that PG is more common in children with CD than UC, and is related mostly to the colonic involvement, presence of erythema nodosum, and arthritis [24].

There is one case report of a nine-year-old boy with a severe form of UC who presented with PG that had primarily been diagnosed as Henoch-Schonlein purpura. The authors emphasize the importance of prompt and correct diagnosis and the collaboration between specialists, as misdiagnosis is often life-threatening [22].

Other less common reactivation skin manifestations of pediatric IBD have been reported, including cutaneous small-vessel vasculitis, necrotizing vasculitis, leukocytoclastic vasculitis, and cutaneous polyarteritis nodosa [30]. 

Butts et al. reported a case of a 12-year-old girl with an acute episode of UC who developed an ecchymotic rash at the site of the intravenous catheter that was later on diagnosed as leukocytoclastic vasculitis [31]. Ho et al. reported two cases of cutaneous small-vessel vasculitis in children with UC; in these cases, the IBD diagnosis should be considered only after excluding more common causes such as immunoglobulin A vasculitis, drug-induced vasculitis, or infections [39].

Polyarteritis nodosa (PN) is a rare vasculitis in childhood with three forms of presentation: infantile (which is now recognized as a severe form of Kawasaki disease), cutaneous, and systemic. Cutaneous PN affects vessels of the deep skin and the subcutaneous tissue, manifesting as subcutaneous nodules, livedo reticularis, and ulcerations located mainly on legs [64]. Although in young adults cutaneous PN has been linked to CD, this connection during childhood has not been well documented to this moment.

A rare dermatological manifestation in pediatric IBD is Sweet’s syndrome. It manifests with painful erythematous papules, plaques, and nodules located on the arms, limbs, trunk, and face; it is usually associated with the disease level of activity. Sweet’s syndrome has been reported at various ages, including multiple pediatric patients and neonates, but it has been linked mostly to leukemia or connective tissue diseases and only occasionally to IBD [4]. Kim MJ et al. described a case of Sweet’s syndrome on a pediatric patient that had just begun treatment with azathioprine for UC for 10 days [33].

### 3.3. Manifestations Related to Malabsorption or Treatment

Treatment for IBD includes a series of classic pharmacological agents but new medications are being developed at the moment. Over the past years, drugs used for pediatric IBD patients have been incriminated for a series of adverse effects, some of which have dermatological implications [65].

The use of anti-TNF agents on a larger scale in IBD patients has led to a higher rate of adverse effects, including early-onset local reactions (up to four hours from administration), late-onset local reactions (4 hours to 14 days from administration), opportunistic skin infections, psoriasiform lesions, and a potentially increased risk for skin cancer [1].

The authors report that Infliximab was the anti-TNF agent incriminated for most dermatological complications, associated in particular with the development of psoriasiform lesions (40% of these patients required a change in medication-dose or interval change, a different anti-TNF agent, or a switch to another medication class) [32,66]. Also, additional risk factors for skin lesions were female gender and smoking [67].

No significant difference was noted between children with CD or UC regarding the anti-TNF agents’ cutaneous adverse effects [67].

In pediatric IBD patients treated with biological agents, psoriasis presents with crusting and weeping as compared to a classic psoriatic eruption which appears as dry plaques. It is located mostly on the scalp, sometimes evolving into severe alopecia, and skin folds (retro auricular and umbilical areas). The psoriasis onset may be at any moment after anti-TNF agents’ initiation, but IBD activity appears to be in remission at the time of the onset.

Skin infections and eczemas are the second most cited cutaneous side effects in pediatric IBD patients treated with anti-TNF agents. Less frequent dermatological manifestations are cited as well, such as type IV hypersensitivity reactions, lupus-like photosensitivity, urticaria, hidradenitis suppurativa, alopecia areata, granuloma annulare, and acrocyanosis [34].

In one retrospective study, Courbette et al. highlighted that the median time of paradoxical psoriasis appearance in children treated with Infliximab was after the eighth infection which corresponded to 355 days (from the moment of treatment initiation). In this study, psoriasis occurred in CD patients only, and it was associated with perianal lesions suggesting that perianal lesions represent a risk factor for psoriasis in CD patients [35].

One study on mortality and cancer appearance in pediatric-onset IBD showed that 1.3% of people developed a type of cancer after a 15-year follow-up (9 patients out of 698), and two of these patients developed basal cell carcinoma. Almost half of the patients who developed a form of cancer (four out of nine) had received immunosuppressants or anti-TNF medications [36].

Deneau et al. reported a case of Epstein–Barr virus-driven malignancy while receiving Infliximab in an 11-year-old-boy presenting with IBD, periodic fever, aphthous stomatitis, and cervical adenitis [37].

More rare cutaneous adverse effects have been reported as well. Rosenbaum et al. published a case report where they underline the association of DRESS (drug rash and eosinophilia with systemic symptoms) and sulfasalazine treatment in an 11-year-old patient with IBD [38].

Patients on biologic therapies should be counseled on the possible cutaneous side effects and the importance of skin protection. One study highlighted the fact that out of 169 IBD patients receiving biologic/immunosuppressive treatments, only 4% benefited from proper counseling on possible dermatologic side effects and primary prevention strategies [68].

Viola et al. suggested that before the initiation of biologics, screening for nasal colonization by *Staphylococcus aureus* should be made in order to identify patients at risk for skin and soft tissue infections that could otherwise evolve into systemic complications [69].

Cutaneous manifestations of IBD secondary to malnutrition and/or malabsorption refer to lesions appearing as a result of vitamin or nutrient deficiency such as zinc deficiency (acrodermatitis enteropathica), niacin deficiency (pellagra), vitamin C deficiency (scurvy), vitamin K deficiency (purpuric lesions), and vitamin B deficiency (glossitis, angular cheilitis). Dry skin and unspecified eczema may appear secondary to essential fatty acid deficiency; hair and nail abnormalities may appear secondary to amino acid and protein malabsorption [63,70].

Vitamin A deficiency is classically associated with reversible night blindness but there is a dermatological involvement as well, characterized by xerosis, non-specific desquamation lesions, abnormal keratinization (follicular hyperkeratosis-phrynoderma), and sparse or dry hair [71]. Da Rocha et al. report a case of vitamin A deficiency associated with night blindness in a patient with complicated CD [72].

Acrodermatitis enteropathica (Zinc deficiency) manifests as red, inflamed areas of dry skin (that may evolve into pus containing lesions), hair loss, or oral ulcers. Zinc deficiency should be considered in IBD patients especially in cases of skin lesions of unknown etiology [73].

Chinese authors reported one case of pellagra in a patient with known CD. In developed countries, pellagra is rarely reported, and physicians may be tempted to overlook this diagnosis or to associate it only with poor socio-economic status. Pellagra is characterized by the three “D’s” triad: dermatitis, diarrhea, and dementia; left untreated, death, the fourth “D”, may appear [74]. Cutaneous manifestations include erythematous desquamating rash with surrounding hyperpigmentation [75].

Lesions of immune thrombocytopenic purpura (ITP) have been sporadically reported in association with IBD cases, mostly UC. The pathogenesis behind this association has not been established, but the treatment regime for UC seems to also control the ITP. Funato et al. reported the case of a 12-year-old girl who presented with ITP and associated symptomatology of IBD; she was later diagnosed with UC and successfully treated with prednisolone and 5-aminosalicylic acid [47].

One topic of paramount importance in the management of pediatric patients with IBD is iron deficiency. In a study group of 75 pediatric IBD patients, iron deficiency was found in 58.6% of children with CD and 71.7% of children with UC; out of all patients with iron depletion, 70.4% had active disease and 57.1% were in remission. Iron deficiency may present with dermatological signs such as alopecia, glossitis, non-specific rash, and stomatitis [46].

## 4. Discussions

The physiopathology behind the extraintestinal manifestation of pediatric IBD is not well understood, but authors have incriminated an immunologically mediated mechanism. One hypothesis refers to the antigen cross-reactivity shared by extraintestinal organs. These manifestations seem to be associated with genes in the HLA region (uveitis, primary sclerosing cholangitis, and ankylosing spondylitis) [48,76].

There are also non-genetic factors promoting the appearance of extraintestinal manifestations of IBD in children, such as malabsorption, protein-losing enteropathy, intestinal resections, leading to nutrient, protein, and vitamin deficiencies. Interestingly, it seems that the presence of one extraintestinal manifestation predisposes to the development of additional extraintestinal manifestations [59].

Dermatological manifestations are not uncommon in pediatric IBD patients. There are specific signs such as fissures, fistulas, aphthous stomatitis, and pyostomatitis vegetans which are known to be highly associated with active IBD, but there are also non-specific lesions such as aphthous stomatitis, EN, PG, Sweet’s syndrome, and vasculitis that may be found in otherwise healthy subjects and may represent the first sign of the underlying intestinal disease [77].

MCD diagnosis in children encounters some particularities, especially in the absence of digestive symptoms, mostly because of the distribution of lesions on the genital area, which may be considered as contact dermatitis, candidiasis, and even sexual abuse. When MCD is considered in children, the literature suggests screening for anorectal strictures, as they seem more common in the pediatric population presented with MCD [18].

The pathophysiology behind the association of EBA and IBD is yet to be established. The isotypes of IgG autoantibodies targeted against type VII collagen show a different distribution pattern in EBA and IBD, suggesting that the targeted epitopes on type VII collagen are different in EBA and IBD (this could explain the absence of skin-blistering in a vast majority of IBD patients) [28].

For specific lesions, treatment of the underlying disease is essential and may be curative, but in selected cases topical and/or systemic agents may be effective as well. In pyostomatitis vegetans, the treatment of choice appears to be systemic corticotherapy but authors reported successful management of pyostomatitis vegetans using azathioprine or cyclosporine A [78].

EN is the most common form of panniculitis, an inflammatory process of subcutaneous fat lobules causing tender symmetrical erythematous nodules on the extensor surfaces of the lower limbs. It appears as an immunologic response to a variety of different antigens and has been closely linked to IBD activity [19,27].

Infectious diseases (streptococcal and Epstein–Barr virus infections) and IBD are the most common cause of EN among children from developed countries, whereas in developing countries EN has been associated mostly with tuberculosis. However, up to 60% of cases remain idiopathic [25].

There are four major clinical variants of PG: the classical one (ulcerative lesions), bullous lesions, pustular appearance, and the vegetative form, but there have been reports of subtypes of peristomal and superficial granulomatous lesions, each variant transitioning from one to another [24].

In contrast to EN, PG tends to present a more severe course and be more resistant to classic therapy (wound care, topical agents, and antibiotics in cases of infections), sometimes necessitating aggressive therapeutic strategies [79].

There are only two randomized controlled trials on adult subjects and none in the pediatric population regarding the treatment for PG. The authors suggest that treatment should include prompt therapeutic strategies because of the debilitating nature of the disease. Local or topical drug applications or injections (intralesional corticosteroid injection, topical sodium cromoglycate, and 5-aminosalicylic acid) have been suggested for mild cases. There are case reports of children who responded well to oral corticosteroids, immunomodulators, and anti-TNF-α (anti-tumor necrosis factor) agents [62,80].

Numerous therapeutic strategies are nowadays available for pediatric IBD patients, such as corticosteroids, 5-aminosalicylic acid, immunosuppressants, and biological agents. It is not unexpected that the vast therapeutic options may lead to important side effects such as dermatological ones which have an important impact on patients’ self-esteem and may lead to a breach of trust regarding the treatment [77].

The pathophysiology behind the paradoxical appearance of psoriasis is not well understood: anti-TNF agents induce the activation of autoreactive T cells and increase interferon-α activity, leading to high levels of pro-inflammatory cytokines (interleukin 12, 17, and 23). Also, it seems that polymorphism in the IL-23R gene may have a role in the development of paradoxical infliximab-induced psoriasis. The association between psoriasis and perianal lesions in CD patients has not been established but it seems that they share a cutaneous immune response [35].

On the other hand, it appears that fecal calprotectin has significantly lower levels in IBD patients with dermatological manifestations and anti-TNF treatment [34].

Cutaneous lesions appearing secondary to IBD treatment usually benefit from topical therapies or immunomodulators. However, if these strategies fail to control the lesions, a change of class medication may be taken into consideration.

Melanoma and non-melanoma skin cancers have been linked to IBD treatment, including thiopurines and anti-TNF agents. Special efforts should be put into prevention strategies and proper counseling [81].

Adolescents are at a period of particular psychiatric vulnerability, a time of brain maturation and behavioral changes. Teenagers with IBD seem to present a higher risk of developing depression in comparison to adults or pediatric patients with other chronic diseases [82,83]. Dermatological lesions have an important impact on adolescents’ self-confidence and perception of self-image, sometimes triggering feelings of embarrassment with a negative impact on self-esteem. Studies exploring the change in depression over time in groups of pediatric IBD patients have shown that cognitive behavioral therapy may reduce depression and may improve quality of life in this particular population [84].

Complementary and alternative medicines have been studied for pediatric IBD patients with several positive effects identified. Studies show that nutritional supplements, special diets (milk or dairy-free, low carbohydrates, gluten-free) improved the patients’ self-reported overall health [85].

Alternative medicines are used at high rates in children with chronic diseases, especially diseases which may alter the adolescents’ image. It appears that herbal treatments are preferred, and parents and patients do not always share this information with their physician. Proper counseling and visits to a specialized psychologist remain mandatory in these situations so the teenagers do not interrupt the indicated medication for the underlying disease [86].

Adolescents with IBD face multiple changes at this age; on one hand, there is other’s perception of themselves, of their chronic illness, symptomatology, acute flares, extraintestinal manifestations, and treatment regime. On the other hand, they face a transition period from the pediatric team (pediatric gastroenterologist, dermatologist, general practitioner, and psychologist) to the adult-destined healthcare system where they have to collaborate with a different team. This transition period can be challenging, especially if it involves teenagers prone to anxiety, with multiple debilitating scar or inflammatory lesions.

The strength of our review is that it contributes to the limited literature on the pediatric IBD population with dermatological manifestations. Our review highlights not only the most commonly encountered dermatological manifestations in pediatric IBD patients but also the ones rarely encountered in everyday practice and provides an insight into the published authors’ experiences. Further studies remain mandatory in order to generate guidelines and treatment recommendations that would assist the clinicians in handling extraintestinal and more precisely dermatological manifestations in pediatric IBD. 

## 5. Conclusions

There is a high incidence of extraintestinal manifestations of pediatric IBD, especially the dermatological ones. Some cutaneous lesions correlate with the IBD activity and should be given special attention because of their potential debilitating course.

All pediatric patients should be closely examined for any cutaneous or mucosal lesions which may be correlated with systemic diseases especially since a wide part of these lesions appear before the gastrointestinal symptomatology or concomitant with a disease activity flare. Idiopathic EN, PG, or pyostomatitis vegetans should be closely monitored in order to diagnose a possible underlying IBD at the debut. Drugs’ adverse effects should be taken into consideration and proper personalized therapeutic strategies should be considered.

Improved knowledge of these forms of presentation is of paramount importance since they may orientate towards the correct diagnosis or may predict the disease course of activity. General practitioners should perform a rigorous examination of skin and mucosa on all pediatric IBD patients in order to establish an early diagnosis or raise the suspicion of a dermatological manifestation of IBD; furthermore, patients should be referred to a multidisciplinary team (pediatric gastroenterologist and dermatologist) for proper diagnosis and management.

The multidisciplinary team should take into consideration the social and psychological implications of IBD, dermatological manifestations, and IBD complications at such young ages and should recommend psychological counseling when necessary.

## Figures and Tables

**Table 1:** Classification of dermatological manifestations of pediatric IBD (inflammatory bowel disease).

Specific lesions	Fissures and fistulas, aphthous stomatitis, pyostomatitis vegetans, metastatic Crohn’s disease, epidermolysis bullosa acquisita
Reactivation lesions	Erythema nodosum, pyoderma gangrenosum, aphthous stomatitis, necrotizing vasculitis, cutaneous polyarteritis nodosa, Sweet’s syndrome
Manifestations related to malabsorption or treatment	Acrodermatitis enteropathica, scurvy, purpura, pellagra, stomatitis-glossitis-angular cheilitis, paradoxical psoriasis, abnormal hair and nails
Miscellaneous lesions	Vitiligo, psoriasis, secondary amyloidosis, bowel associated dermatosis-arthritis syndrome

**Table 2:** Types of studies and samples related to dermatological manifestations in pediatric IBD.

Author, Year of Publication	Country	Study Design	Number of Patients	Reported Dermatological Manifestations of Pediatric IBD		
				Specific Lesions	Reactivation Lesions	Lesions Related to Malabsorption/Medication
Jose et al., 2009 [3]	USA	Retrospective	1649	Aphthous stomatitis (*n* = 53)	EN (*n* = 21); PG (*n* = 6)	Psoriasis (*n* = 2)
Jang et al., 2019 [4]	South Korea	Review	-	MCD, epidermolysis bullosa, erythema elevatum iutinum	EN, PG, Sweet’s Syndrome, polyarteritis nodosa	Psoriasis, alopecia
Levine et al., 2011 [5]	USA	Review	-	Oral aphthous stomatitis, genital ulcers, MCD,	EN, PG, Sweet’s syndrome	Psoriasis, eczematous lesions
Stawarski et al., 2006 [7]	Poland	Retrospective	184	Perianal changes, external intestinal fistulae		
Mantegazza et al., 2016 [9]	Italy	Review	-	Pyostomatitis vegetans, oral aphthae		Glossitis, non-specific gingivitis, stomatitis, cheilitis, mucosal ulcers, lichen planus, diffuse pustules
Ribaldone et al., 2020 [10]	UK, Italy	Review	-	Deep ulcerations, lip swelling, fissures, cobblestoning, aphthous stomatitis, pyostomatitis vegetans,		Angular cheilitis, mucogingivitis
Cohen et al., 2020 [11]	Israel	Retrospective	100	Aphthous stomatitis		
Lankarani et al., 2013 [12]	Iran	Review	-	Cobblestoning, lip swelling, fissures, aphthous stomatitis, perioral erythema with scaling, pyostomatitis vegetans,		Mucogingivitis, angular cheilitis
Trost et al., 2005 [13]	USA	Review	-	Aphthous stomatitis, cobblestoning, pyostomatitis vegetans	PG, EN	
Pazheri et al., 2010 [14]	USA	Case Report	1	Pyostomatitis vegetans		
Femiano et al., 2009 [15]	Italy	Review	-	Pyostomatitis vegetans		
Huang et al., 2012 [16]	USA	Review	-	Perianal lesions, cobblestoning of the buccal mucosa, aphthous stomatitis, ulcers, pyodermatita vegetans, pyostomatita vegetans, acquired epidermolysis bullosa	EN, PG, neutrophilic dermatoses, leukocytoclastic vasculitis	Acrodermatitis enteropathica, pellagra, scurvy, purpura, xeroderma, unspecified eczema, hair loss, lichen planus, erythema multiforme, bullous dermatosis, Stevens–Johnson syndrome, acne, psoriasis
Blasco-Alonso et al., 2016 [17]	Spain	Case report	4	MCD		
Bender-Heine et al., 2017 [18]	USA	Review	-	MCD		
Lee et al., 2016 [19]	South Korea	Retrospective	73	Perianal abscesses and/or fistulas (*n* = 37), vulvitis (*n* = 1), anal skin tags (*n* = 25)	EN (*n* = 2)	
Korelitz et al., 2010 [20]	USA	Review	-	Anal skin tags		
Keljo et al., 2009 [21]	USA	Retrospective	276	Perianal lesions (*n* = 41), skin tags and fissures (*n* = 13), fistulas and/or abscesses (*n* = 28)		
Kierkuś et al., 2014 [22]	Poland	Case Report	1		PG	
Schoch et al., 2017 [23]	USA	Retrospective	13		PG	
Manda et al., 2017 [24]	Malawi	Case report	1		PG	
Weinstein et al., 2005 [25]	USA	Case report	1		EN	
Dotson et al., 2010 [26]	USA	Prospective	1009	Aphthous stomatitis, perianal disease	EN	
Schwartz et al., 2007 [27]	USA	Review	-		EN	
Reddy et al., 2013 [28]	UK	Review	1969	Epidermolysis bullosa acquisita		
Russo et al., 2015 [29]	Italy	Case report	1	Epidermolysis bullosa acquisita		
Simonetti et al., 2015 [30]	Italy	Review	-		cutaneous vasculitis	
Butts et al., 2014 [31]	USA	Case report and literature review	1	Dermatitis herpetiformis, epidermolysis bullosa acquisita	EN, PG, Sweet’s syndrome, necrotizing vasculitis, leukocytoclastic vasculitis	Psoriasis
Sridhar et al., 2018 [32]	USA	Retrospective	409			Infections (*n* = 28), psoriasis (*n* = 33), eczema (*n* = 10
Kim et al., 2011 [33]	South Korea	Case report	1			Sweet’s syndrome 10 days after treatment with azathioprine
Cossio et al., 2020 [34]	Canada	Retrospective	343			Psoriasiform eruptions (*n* = 20); skin infections (*n* = 10); eczematous eruptions (*n* = 5); type IV hypersensitivity reactions (*n* = 3), lupus-like photosensitivity (*n* = 1), urticaria (*n* = 1), alopecia areata (*n* = 1), hidradenitis suppurativa (*n* = 1), pyoderma gangrenosum (*n* = 1), granuloma annulare (*n* = 1), acrocyanosis (*n* = 1)
Courbette et al., 2019 [35]	France	Retrospective	147			Infliximab-induced psoriasis (*n* = 20)
Peneau et al., 2013 [36]	France	Retrospective	698			Basal cell carcinoma (*n* = 2)
Deneau et al., 2010 [37]	USA	Case report	1	Aphthous stomatitis	Hemophagocytic lymphohistiocytosis, Epstein-Barr virus-positive natural killer T-cell lymphoma	
Rosenbaum et al., 2010 [38]	Australia	Case report	1			Drug rash and eosinophilia with systemic symptoms (DRESS) secondary to sulfasalazine
Ho et al., 2017 [39]	USA	Case report and literature review	2		Cutaneous small-vessel vasculitis	
Toussi et al., 2013 [40]	USA	Systematic literature review	-			Abscess/cellulitis (*n* = 8) secondary to anti TNF agents
Sherlock et al., 2013 [41]	Canada	Retrospective	172			Psoriasis or psoriasiform skin lesions secondary to Infliximab
Nuti et al., 2014 [42]	Italy	Retrospective observational	78			Infusion reactions, psoriasis (*n* = 9), minor infections (herpes simplex infections, oral candidiasis, folliculitis)—secondary to biological therapy
Savasan et al, 2013 [43]	USA	Case report	1			Hidradenitis suppurativa secondary to Infliximab
McCluggage, 2011 [44]	USA	Review	-			Infusion or injection-site reactions secondary to anti-TNF agents
Bradley et al., 2012 [45]	USA	Review	-			Rashes (Thiopurine immunomodulators), hirsutism (Calcineurin inhibitors)
Krawiec et al., 2020 [46]	Poland	Observational	-			Alopecia, stomatitis, glossitis secondary to iron deficiency
Funato et al., 2011 [47]	Japan	Case Report	1			Multiple purpuric lesions
Aloi, 2009 [48]	Italy	Review	-	Perianal skin tags, oral aphthous ulcers, MCD	EN, PG	
Keyal et al., 2018 [49]	China	Review	-	Perianal and oral lesions, MCD	EN, PG, Sweet’s syndrome, Bowel-associated dermatosis-arthritis syndrome, Pyodermatitis-pyostomatitis vegetans, SAPHO syndrome (acne conglobata or fulminans, pustulosis, hidradenitis suppurativa, dissecting cellulitis of the scalp)	Phrynoderma, stomatitis-glossitis-angular cheilitis, scurvy, seborrheic-type dermatitis, bruising, petechiae, dry skin, eczema, slow wound healing, hair hypopigmentation, glossitis, nail abnormalities, acrodermatitis enteropathica, eczematous and psoriasiform skin eruptions, skin and soft tissue infections
Zippi et al., 2013 [50]	Italy	Review	-		EN, PG	Paradoxical psoriasis
Evans et al., 2007 [51]	USA	Review	-	Aphthous stomatitis	EN, PG	
Shan et al., 2019 [52]	China	Retrospective	161	Aphthous ulcer (*n* = 39), skip lesions (*n* = 19), perianal disease		
Al-Mendalawi et al, 2018 [53]	Saudi Arabia	Retrospective	66		EN, PG	
Navallo et al, 2017 [54]	USA	Case series	5			paradoxical psoriasis secondary to Infliximab (*n* = 3) and Adalimumab (*n* = 1), eczema, pityriasis rosea
Bukhari et al., 2015 [55]	Saudi Arabia	Case report	1			Infliximab-induced psoriasis
Greuter et al., 2017 [56]	Switzerland	Retrospective	329	Aphthous stomatitis	EN, PG	Psoriasis
Guariso et al., 2010 [57]	Italy	Retrospective	133	Perianal disease (*n* = 17), oral aphthous lesions (*n* = 4)	EN (*n* = 4)	
Andrisani et al., 2012 [58]	Italy	Review	-		EN, PG	
Woo et al., 2015 [59]		Case report	1	Mucosal erythema, mucosal-colored swellings, and ulcerations of the gingiva, nodular swelling of the interdental papillae, tissue tags, polyps, aphthous ulcerations, perioral dermatitis		Angular cheilitis, stomatitis, glossitis
Rizvi et al, 2019 [60]	USA	Case report	1			Cutaneous ulcerations secondary to prolidase deficiency in a toddler with very-early-onset CD
Ben Rabeh et al, 2019 [8]	Tunisia	Retrospective	14	Ulcerative skin eruption	EN	vitiligo
Molnar et al, 2011 [61]	Hungary	Case report	1	Skin tags, swelling of the lips, oral mucosa, pyostomatitis vegetans		

EN: Erythema nodosum; PG: Pyoderma gangrenosum; MCD: Metastatic Crohn’s disease.
